# Dominance of the Unaffected Hemisphere Motor Network and Its Role in the Behavior of Chronic Stroke Survivors

**DOI:** 10.3389/fnhum.2016.00650

**Published:** 2016-12-27

**Authors:** Sahil Bajaj, Stephen N. Housley, David Wu, Mukesh Dhamala, G. A. James, Andrew J. Butler

**Affiliations:** ^1^Department of Physics and Astronomy, Georgia State University, AtlantaGA, USA; ^2^Department of Psychiatry, College of Medicine, University of Arizona, TucsonAZ, USA; ^3^Byrdine F. Lewis School of Nursing and Health Professions, Georgia State University, AtlantaGA, USA; ^4^Joint Center for Advanced Brain Imaging, Center for Behavioral Neuroscience, Center for Nano-Optics, Center for Diagnostics and Therapeutics, Georgia State University, AtlantaGA, USA; ^5^Neuroscience Institute, Georgia State University, AtlantaGA, USA; ^6^Psychiatric Research Institute, University of Arkansas for Medical Sciences, Little RockAR, USA; ^7^Department of Veterans Affairs, Atlanta Rehabilitation Research and Development Center of Excellence, DecaturGA, USA

**Keywords:** functional magnetic resonance imaging (fMRI), effective connectivity, dynamic causal modeling (DCM), motor task, stroke, affected hemisphere, unaffected hemisphere

## Abstract

Balance of motor network activity between the two brain hemispheres after stroke is crucial for functional recovery. Several studies have extensively studied the role of the affected brain hemisphere to better understand changes in motor network activity following stroke. Very few studies have examined the role of the unaffected brain hemisphere and confirmed the test–retest reliability of connectivity measures on unaffected hemisphere. We recorded blood oxygenation level dependent functional magnetic resonance imaging (fMRI) signals from nine stroke survivors with hemiparesis of the left or right hand. Participants performed a motor execution task with affected hand, unaffected hand, and both hands simultaneously. Participants returned for a repeat fMRI scan 1 week later. Using dynamic causal modeling (DCM), we evaluated effective connectivity among three motor areas: the primary motor area (M1), the premotor cortex (PMC) and the supplementary motor area for the affected and unaffected hemispheres separately. Five participants’ manual motor ability was assessed by Fugl-Meyer Motor Assessment scores and root-mean square error of participants’ tracking ability during a robot-assisted game. We found (i) that the task performance with the affected hand resulted in strengthening of the connectivity pattern for unaffected hemisphere, (ii) an identical network of the unaffected hemisphere when participants performed the task with their unaffected hand, and (iii) the pattern of directional connectivity observed in the affected hemisphere was identical for tasks using the affected hand only or both hands. Furthermore, paired *t*-test comparison found no significant differences in connectivity strength for any path when compared with one-week follow-up. Brain-behavior linear correlation analysis showed that the connectivity patterns in the unaffected hemisphere more accurately reflected the behavioral conditions than the connectivity patterns in the affected hemisphere. Above findings enrich our knowledge of unaffected brain hemisphere following stroke, which further strengthens our neurobiological understanding of stroke-affected brain and can help to effectively identify and apply stroke-treatments.

## Introduction

An estimated 795,000 Americans suffer a stroke annually, leading to long-term disability for an estimated 6.4 million Americans. Many stroke survivors exhibit some degree of motor impairment that limits functional status after stroke. Advances in acute care medicine have significantly reduced mortality, which has coincidentally led to rising numbers of stroke survivors that utilize rehabilitation therapies. As the body of evidence of stroke rehabilitation is expanding (Dobkin, 2004; Brewer et al., 2013; Bajaj et al., 2015b), it has become exceedingly important to explore how brain networks are influenced following stroke and the role those networks play in functional recovery. A rich neurobiological understanding of the basic principles of stroke-recovery will aid in the development of more effective stroke treatments.

Over the past several years, numerous studies have been proposed to better understand the connectivity patterns in motor network of people suffering from stroke. Most of the studies have focused on the basic motor networks directly involved after stroke (before and after stroke treatment) and compared the results with healthy controls. The primary motor area (M1), which is an integral part of basic motor network, due to its association with upper-limb recovery, is the most common target for stroke therapies. Other motor areas such as premotor cortex (PMC) and supplementary motor area (SMA) are functionally and anatomically in close association with M1 and play a crucial role to execute motor tasks (Bajaj et al., 2014, 2015a,b). Previous studies have discussed the role of the motor network in the unaffected hemisphere of stroke patients and its test–retest reliability with time. Although investigating changes in motor network connectivity strength provide important insight into brain reorganization following stroke, few studies assessing these changes are grounded by the functional ability and motor performance outcomes that are important for stroke survivors with residual upper limb impairment (Fong et al., 2001; Arya et al., 2011; Bajaj et al., 2015a). Recently, in a stroke study, Li et al. (2016) observed significant correlations between the connectivity strength and functional ability, implying that the connectivity of ipsilateral M1 may be useful in evaluating and predicting functional ability and motor performance. This is in agreement with other studies (Grefkes and Fink, 2011; Lindenberg et al., 2012; Chen and Schlaug, 2013) that have found changes in cortical network connectivity of stroke patients are associated with impaired functional ability and motor performance. This is an evolving area of research, with most studies associating clinical outcome to a single region of interest (ROI) association, and fewer studies relating outcome to more complex network models (Park et al., 2011). To our knowledge, no studies have previously compared the role that affected and unaffected hemispheres networks play in encoding stroke patients’ functional ability while simultaneously assessing time-dependent test–retest reliability of these outcomes.

The role of unaffected hemisphere in motor recovery has been considered somewhat controversial (Buetefisch, 2015). It has been reported that the neural substrates in the unaffected hemisphere can mediate recovery only when such substrates in the affected hemisphere are significantly damaged (John et al., 2015). In other studies, abnormalities have been reported in the unaffected arm after stroke, which further depends on whether the infarct was in the dominant or non-dominant hemisphere (Colebatch and Gandevia, 1989; Haaland and Harrington, 1989; Jones et al., 1989; Winstein and Pohl, 1995; Haaland et al., 2004). It is hypothesized that the behavioral recovery observed after stroke is supported by the sensorimotor network in the affected hemisphere (Pineiro et al., 2002; Loubinoux et al., 2003; Calautti et al., 2007; Loubinoux, 2007), whereas it is also hypothesized that the unaffected hemisphere may support motor-recovery (O’Shea et al., 2007; Riecker et al., 2010; Rehme et al., 2011). Although a significant ipsilateral activation has been considered as a marker for poor motor recovery (Ward et al., 2003) alternatively, this has been found in motor areas of subacute and chronic stroke patients (Weiller et al., 1992; Seitz et al., 1998; Bütefisch et al., 2005; Lotze et al., 2006; Schaechter and Perdue, 2008).

Reliability of functional and effective connectivity among motor areas and reliability of various neuroimaging tools over time has been another important aspect to consider when assessing cortical mechanism of recovery. The reliability of functional MRI (fMRI) during visual motor tasks in stroke patients has been tested within and between sessions. By comparing interclass correlation coefficients (ICC), within-session reliability has been reported to be higher than between session reliability, but the overall results reflect that brain activations are reproducible and such research designs could be used for stroke patients (Kimberley et al., 2008b). Using ROI seed-based and ROI correlation matrix approaches, a 1-year test–retest reliability of intrinsic connectivity network was confirmed for older adults using fMRI (Guo et al., 2012). This study was found to be consistent with other short-term reliability studies on young (Schwarz and McGonigle, 2011) as well as older controls (Telesford et al., 2010).

In order to better understand the brain connectivity pattern of the affected and unaffected hemispheres while performing the motor execution task, nine stroke survivors underwent fMRI scanning over two sessions with one-week separation. Our goals in this study were to: (a) Explore the brain connectivity pattern for: (i) affected hemisphere during tapping with affected hand only *(AHem-aHand)* (ii) affected hemisphere during tapping with both hands (affected and unaffected) simultaneously *(AHem-bHand)* (iii) unaffected hemisphere during tapping with affected hand only *(UHem-aHand)*, and (iv) unaffected hemisphere during tapping with unaffected hand only *(UHem-uHand)*; (b) check if bilateral tapping (i.e., tapping with both hands) strengthened the connectivity patterns more in affected hemisphere compared to unilateral tapping (i.e., tapping with affected hand only) *(AHem-bHand vs. AHem-aHand)*; (c) check if unilateral tapping with unaffected hand better estimated the connectivity pattern on unaffected hemisphere *(UHem-uHand)* than the connectivity pattern on affected *(AHem-aHand)* and unaffected *(UHem-aHand)* hemispheres while tapping with affected hand; (d) check if brain connectivity parameters were reliable between two sessions of one week apart; and (e) explore the brain-behavior correlations for affected and unaffected hemispheres.

We hypothesized that the:

(1) connectivity pattern would be (a) stronger for *AHem-bHand* than *AHem-aHand* (b) stronger for *UHem-uHand* than for either *AHem-aHand* or *AHem-bHand* and (c) weaker and different for *UHem-aHand* than *AHem-aHand*.
(2) connectivity strength parameters would significantly (a) positively correlate with FMA scores and (b) negatively correlate with RMSE scores for *UHem-uHand* only.

Here higher FMA scores and lower RMSE scores represent better performance and vice-versa.

## Materials and Methods

### Participants

Stroke survivors between the ages of 45 and 90 with a moderate to severe unilateral ischemic stroke were recruited. Inclusion criteria included persistent hemiparesis as indicated by a score of 1–3 on the motor arm item of the NIH Stroke Scale (Brott et al., 1989) and significant impairment that limited their activities of daily living (ADL). Those with clinically significant comprised mental status within three days of enrollment were excluded. A total of nine participants were enrolled, all of them had heterogeneous stroke locations distributed to either the left (*n* = 5) or right hemispheres (*n* = 4), resulting in hemiparesis of the contralateral side. Participants underwent fMRI of a motor task (described below) during two sessions with a one-week gap between sessions. Data from one participant was excluded from the analysis because the time between sessions was greater than one week. Written consent was obtained from each participant prior to the experiment and all the participants were compensated for their participation and time. The experimental protocol was approved by the Emory Institutional Review Board (IRB).

### Tasks

All the participants were instructed to lie down in the scanner with arms outstretched close to their body. A block-design paradigm (total duration 550 s) was used during the experiment where participants were instructed to tap with their left hand index finger (LH), right hand index finger (RH), and both hand index fingers (BH) for 20 s. The protocol for this study was set in a way that all stroke patients had both their affected and unaffected hemispheres involved during scanning. For baseline, a passive resting state (Rest) condition for 10 s was included (i) before the tapping task, (ii) in between two tapping tasks, and (iii) at the end of the task. The same paradigm was repeated six times as following:

(i) Rest-LH-Rest-RH-Rest-BH (1–90 seconds)
(ii) Rest-RH-Rest-LH-Rest-BH (91–180 s)
(iii) Rest-LH-Rest-BH-Rest-RH (181–270 s)
(iv) Rest-RH-Rest-BH-Rest-LH (271–360 s)
(v) Rest-BH-Rest-LH-Rest-RH (361–450 s)
(vi) Rest-BH-Rest-RH-Rest-LH (451–540 s)
(viii) Rest (541–550 seconds)

### Behavioral Data

In addition to imaging data, clinical data was also collected from five of the nine stroke survivors (clinical data was unavailable on the remaining four). Blinded evaluators assessed clinical outcome measures. Functional ability of affected hand was assessed by the upper extremity portion of the Fugl-Meyer Motor Assessment (FMA) Scale. This is a 33-item test with each item scored on a 3-point ordinal scale that measures motor function and recovery after stroke (Fugl-Meyer et al., 1975). Scores ranged from 0 (no function) to 66 (normal function) (Sanford et al., 1993). The FMA is a reliable and valid tool for measuring UE impairment following stroke (Gladstone et al., 2002).

To evaluate hand and wrist motor performance of affected hand, root mean squared error (RMSE) was used to determine how closely the participants followed a target presented on a computer monitor, where lower RMSE indicates more accurate tracking and improved motor performance (Wulf and Schmidt, 1997; Gentili et al., 2010). Observed and target tracking performances were recorded (50 Hz sampling rate). Waveform tracking error, as assessed by RMSE, was calculated for the first sine waveform by comparing the observed tracking to the target tracking (equation 1), where *y*_i_ is the observed tracking and *ŷ*_i_ is the target tracking at time/place *i* for *n* observations. This was accomplished by sampling approximately the same length block (first 5 min) for every participant. The sampling method was chosen to reduce the impact of fatigue on performance following repeated training bouts (Lorist et al., 2002) and attenuate the occurrence of experience-dependent plasticity within the cortical-cerebellar and cortical-striatal neural systems during the fast learning phase (Doyon and Benali, 2005). The first 40-s of each block were discarded to account for system delay and allow the participant to reach a steady-state tracking performance. The remaining time in the block was used for analysis. The overall RMSE for a given session’s block was calculated using the following formula:

RMSEOverall=Σi=1n(yi^−yi)2n

Participant demographics, time post stroke, stroke locations, and baseline behavioral data (FMA and RMSE scores) of the stroke patients are summarized in **Supplementary Table S1**.

### Imaging

MR imaging was performed at Wesley Woods Center of Emory University, Atlanta, GA, USA. Images were acquired with a Siemens 3.0 T Magnetom Trio scanner (Siemens Medical Solutions, Malvern, PA, USA) using a standard quadrature head coil and multi-band sequence (TR/TE/FA = 1000 ms/30 ms/65°, 550 measurements for total duration 9 min10 s, resolution = 3 mm × 3 mm × 3 mm and 52 transversal slices). An anatomical image of each participant was acquired using a 3D magnetization-prepared rapid acquisition gradient echo (MPRAGE) sequence which consisted of 176 sagittal slices of 1 mm-thickness (resolution = 1 mm × 1 mm, in-plane matrix = 256 × 256) with TR/TE/FA = 2300 ms/2.89 ms/8°. Participants repeated the fMRI task six times (scans) per imaging session and underwent two scanning sessions separated by 1 week.

### Data Analysis

#### FMRI Preprocessing

Functional MRI data were preprocessed by using SPM12^1^ (Wellcome Trust Centre for Neuroimaging, London). The preprocessing steps involved slice time correction, realignment, normalization, and smoothing. Motion correction to the middle functional scan was performed within participant using a six-parameter rigid-body transformation. Three translational and three rotational motion-parameters were stored and used as nuisance covariates. The mean of the motion-corrected images was then co-registered to the individual structural image using a 12-parameter affine transformation. The images were then spatially normalized to the Montreal Neurological Institute (MNI) template (Mazziotta et al., 1995) by applying a 12-parameter affine transformation, followed by a non-linear warping using basis functions (Ashburner and Friston, 1999). Images were subsequently smoothed with an 8-mm isotropic Gaussian kernel and the low-frequency drifts in signal were removed using a standard band-pass-filter with a 128 s cutoff.

#### Volumes of Interest (VOIs)

In this study, we considered basic motor areas (M1, PMC) and the SMA for each participant, which are well known to play a significant role during motor tasks. Hence, we defined six volumes of interest (VOIs): bilateral M1, bilateral PMC, and bilateral SMA in SPM12 using the first Eigen-variate of activations within a sphere of 6 mm radius. Before applying first Eigen-variate, VOIs were centered at (-34, -18, 52), (36, -18, 52), (-34, 0, 56), (34, 0, 56), (-6, -6, 58), and (6, 0, 62) in MNI coordinate system for left M1, right M1, left PMC, right PMC, left SMA, and right SMA, respectively (Cárdenas-Morales et al., 2014; Bajaj et al., 2015a). Before defining VOIs, we performed the standard uni-variate analysis but we did not find consistent/significant brain activations throughout the sample as the participants suffered from stroke and some of the participants had serious difficulty in performing the task, causing insignificant brain activations. In addition to that, since our analysis also involved connectivity in the ipsilateral side of the brain, so significant brain activation was not found consistently.

Hence, all the VOIs were defined by extracting mean time-series from the same set of voxels across the participants for each VOI. For that, we avoided any statistical threshold on activity within areas of interest so that extracted and adjusted time-series data remain spatially identical across all the participants (Parker Jones et al., 2013). The participant specific maxima were constrained to lie within twice the width of Gaussian smoothing kernel (Li et al., 2010; Bajaj et al., 2013).

#### Dynamic Causal Modeling (DCM)

In this study, we used random effects Bayesian model selection (RFX BMS) and Bayesian model averaging (BMA) approaches implemented in DCM12 in SPM12a package^1^ (Wellcome Trust Centre for Neuroimaging, London). Here by defining a model space constituting eight models, we computed expected and exceedance probability of each model, along with individual connection strength parameters. DCM is based on dynamical systems theory. Using a set of differential equations, DCM aims to describe how observed brain responses are generated. It estimates the directed connectivity among functionally distinct brain areas by using bilinear approximations to the coupled brain states and further models the influence of external inputs directly to the brain areas or to the connections between functionally distinct areas (Friston et al., 2003).

The task is done by constructing a model space, constituting a set of models where each model represents a set of intrinsic connections among pre-defined VOI modulated by experimental inputs. Using Bayesian model selection (BMS) approach (Penny et al., 2004; Stephan et al., 2009), an optimal model or a winning model is found by calculating model ‘expected posterior probability’ and model ‘exceedance probability’ of each model. Here, model expected posterior probability represents how likely it is that a model generated the data of randomly chosen subject whereas exceedance probability is a measure of degree of belief about a model having a higher posterior probability than the other remaining models (Wasserman, 2000; Stephan et al., 2010). Here, an optimal model represents the best possible combination of intrinsic and modulatory connections among pre-defined VOIs, which best explains how the observed data are generated. Further, in order to infer individual connectivity measures, another approach known as Bayesian model averaging (BMA) (Penny et al., 2010; Stephan et al., 2010) is used, which estimates a weighted average of each parameter of each model. Here, weighting of each parameter depends upon model evidence of each model.

Since the fMRI data was collected from a clinical group (stroke patients) and there was a possibility of inter-subject variability, we employed BMS and BMA using random-effects analysis (RFX) for group-level inferences (Kasess et al., 2010).

### Plan

**FIGURE 1:**
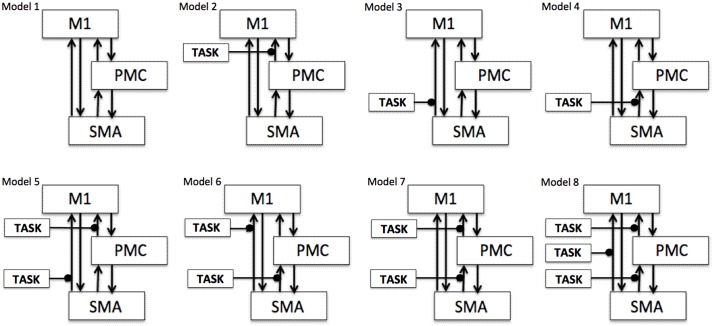
**Specification of model space.** Eight models (models 1–8) are specified subject-wise, constituting bilinear family for both the hemispheres (affected and unaffected) for each analysis (*AHem-aHand, AHem-bHand, UHem-aHand*, *and UHem-uHand*) and for each session. Here ‘TASK’ represents ‘motor execution’ task, performed by either unilateral (left or right) hand or by both hands.

To test our previously stated hypotheses we generated eight models using DCM12^1^, for both hemispheres of all nine participants for each analysis, each session and for each task as shown in **Figure 1** where ‘TASK’ represents tapping with either left hand (LH) (affected/unaffected), right hand (RH) (unaffected/affected), and both hands (BH) (affected and unaffected). Theoretically, total number of models generated was 576 [8 × 9 (participants) × 4 *(AHem-aHand, AHem-bHand, UHem-aHand*, *and UHem-uHand)* × 2 (sessions)]. Since, one subject was excluded from the analysis and the task BH was included only for affected hemisphere, our model space included a total of 512 models [(8 × 8 (participants) × 3 *(AHem-aHand, UHem-aHand, and UHem-uHand)* × 2 (sessions) + (8 × 8 (participants) × 1 *(AHem-bHand)* × 2 (sessions))].

We categorized our connectivity analysis into following analysis:

#### Analysis 1: Connectivity on Affected Hemisphere

##### Analysis 1a: AHem-aHand

This analysis represents the condition when participants tapped with affected hand (right or left) and connectivity strength was calculated between motor areas of the affected hemisphere (left or right) (**Supplementary Figures S1A,B**).

**Supplementary Figure S1A** shows tapping with right affected hand and areas under study are on left hemisphere, which is affected. **Supplementary Figure S1B** shows tapping with left affected hand and right hemisphere brain areas reflect the affected hemisphere.

##### Analysis 1b: AHem-bHand

This analysis represents the condition when stroke survivors tapped with both hands (affected and unaffected) and connectivity strength was calculated between motor areas of the affected hemisphere (**Supplementary Figures S1C,D**).

**Supplementary Figure S1C** shows tapping with both hands (where right hand is affected) and cortical areas under study were focused on the affected- left hemisphere. **Supplementary Figure S1D** shows tapping with both hands (where left hand is affected) and areas under study are on right hemisphere.

#### Analysis 2: Connectivity on Unaffected Hemisphere

##### Analysis 2a: UHem-aHand

This analysis represents the condition when patients tapped with affected hand (right or left) and connectivity strength was calculated between motor areas of the unaffected hemisphere (right or left) (**Supplementary Figures S1E,F**).

**Supplementary Figure S1E** shows tapping with right hand, which is affected, and areas under study are on right hemisphere, which is unaffected. **Supplementary Figure S1F** shows tapping with left hand, which is affected, and areas under study are on left hemisphere, which is unaffected.

##### Analysis 2b: UHem-uHand

This analysis represents the condition when patients tapped with their unaffected hand (left or right) and connectivity strength was calculated between motor areas of the unaffected hemisphere (right or left) (**Supplementary Figures S1G,H**).

**Supplementary Figure S1G** shows tapping with left hand, which is unaffected, and areas under study are on right hemisphere, which is unaffected. **Supplementary Figure S1H** shows tapping with right hand, which is unaffected, and areas under study are on left hemisphere, which is unaffected.

In **Supplementary Figure S1**, affected hemisphere is colored in ‘red’ whereas unaffected hemisphere is colored in ‘gray’.

## Results

### Effective Connectivity: Optimal Model Selection

#### Analysis 1: Connectivity on Affected Hemisphere

##### Analysis 1a: AHem-aHand

**FIGURE 2:**
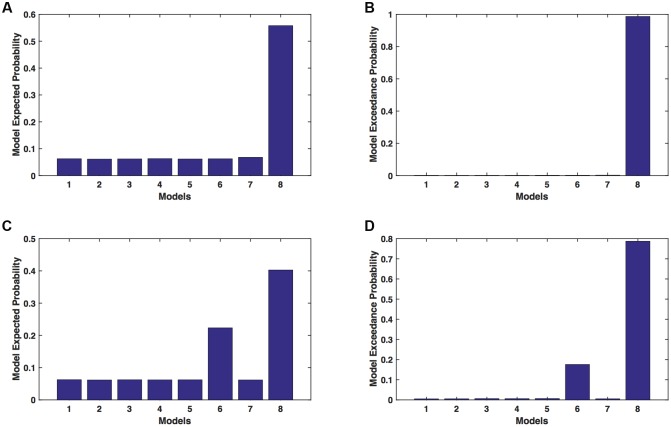
***AHem-aHan*d: Optimal model selection for affected hemisphere.** For *AHem-aHand*, when participants performed the task with affected hand, model expected and model exceedance probabilities are shown for session 1 **(A,B)** and session 2 **(C,D)**.

We calculated model expected (A,C) and model exceedance (B,D) probability for session 1 (**Figures 2A,B**) and for session 2 (**Figures 2C,D**) for networks on affected hemisphere when patients tapped with affected hand. This showed that for *AHem-aHand*, model 8 was dominant with model exceedance probability of (i) 0.987 for session 1 and (ii) 0.787 for session 2. For session 2, model 6 was the second best model with model exceedance probability of 0.175.

##### Analysis 1b: AHem-bHand

**FIGURE 3:**
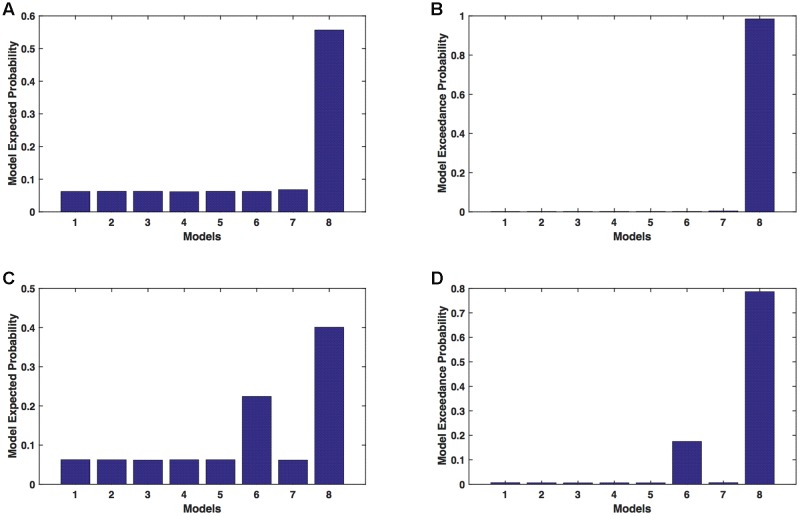
***AHem-bHand:* Optimal model selection for affected hemisphere.** For *AHem-bHand*, when participants performed the task with both (affected and unaffected) hands, model expected and model exceedance probabilities are shown for session 1 **(A,B)** and session 2 **(C,D)**.

We calculated model expected (A,C) and exceedance (B,D) probability for session 1 (**Figures 3A,B**) and for session 2 (**Figures 3C,D**) for networks on affected hemisphere when patients tapped with both (affected and unaffected) hands. The analysis showed that for *AHem-bHand*, model 8 was dominant with model exceedance probability of (i) 0.984 for session 1 and (ii) 0.786 for session 2. For session 2, model 6 was the second best model with model exceedance probability of 0.174.

Here, comparing BMS results for *AHem-aHand* and *AHem-bHand*, we found that model 8 was dominant during during both the sessions for both the analyses. During session 2, both the analyses also showed an identical second best model as model 6.

#### Analysis 2: Connectivity on Unaffected Hemisphere

##### Analysis 2a: *UHem-aHand*

**FIGURE 4:**
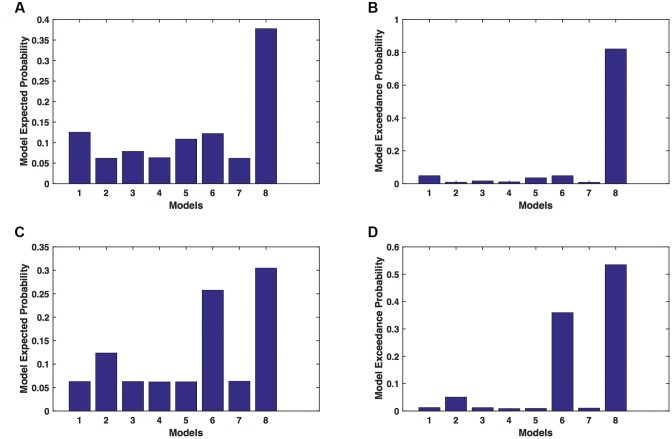
***UHem-aHand:* Optimal model selection for unaffected hemisphere.** For *UHem-aHand*, when participants performed the task with affected hand, model expected and model exceedance probabilities are shown for session 1 **(A,B)** and session 2 **(C,D)**.

We calculated model expected (A,C) and exceedance (B,D) probability for session 1 (**Figures 4A,B**) and for session 2 (**Figures 4C,D**) for networks on unaffected hemisphere when patients tapped with affected hand. This showed that for *UHem-aHand*, model 8 was the dominant with a model exceedance probability of (i) 0.821 for session 1 and (ii) 0.534 for session 2. For session 2, model 6 had model exceedance probability of 0.359.

Here, comparing BMS results for *AHem-aHand* and *UHem-aHand*, we found that although model 8 was dominant during both sessions 1 and 2, the exceedance probability for model 8 decreased from *AHem-aHand* to *UHem-aHand* (from 0.987 to 0.821 for session 1 and from 0.787 to 0.534 for session 2) and increased for model 6 from *AHem-aHand* to *UHem-aHand* (from 0.175 to 0.359 for session 2).

##### Analysis 2b: *UHem-uHand*

**FIGURE 5:**
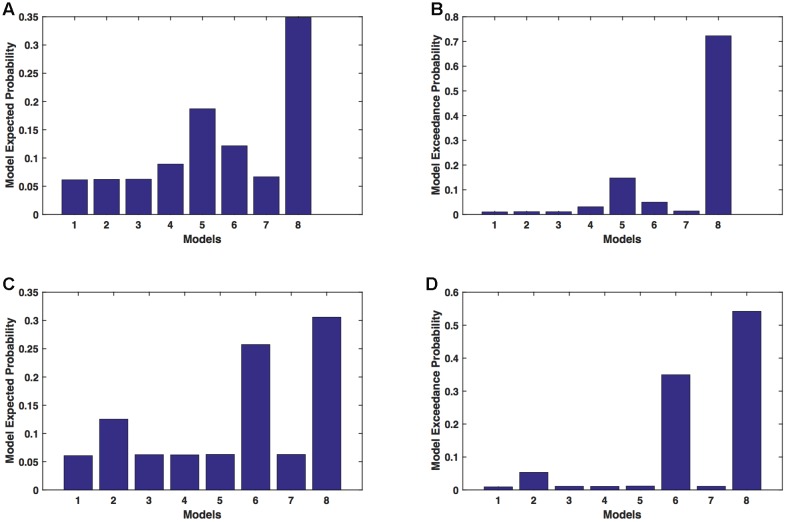
***UHem-uHand:* Optimal model selection for unaffected hemisphere.** For *UHem-uHand*, when participants performed the task with unaffected hand, model expected and model exceedance probabilities are shown for session 1 **(A,B)** and session 2 **(C,D)**.

We calculated model expected (A,C) and exceedance (B,D) probability for session 1 (**Figures 5A,B**) and for session 2 (**Figures 5C,D**) for networks on unaffected hemisphere when patients tapped with unaffected hand. This showed that for *UHem-uHand*, model 8 was dominant with a model exceedance probability of (i) 0.723 for session 1 and (ii) 0.542 for session 2. For session 2, model 6 had model exceedance probability of 0.349.

Comparing BMS results for *AHem-aHand* and *UHem-uHand*, we found that model 8 was dominant during both sessions 1 and 2 but the exceedance probability for model 8 decreased from *AHem-aHand* to *UHem-uHand* (from 0.987 to 0.723 for session 1 and from 0.787 to 0.542 for session 2) and increased for model 6 from *AHem-aHand* to *UHem-uHand* (from 0.175 to 0.349 for session 2).

When BMS results were compared for *UHem-aHand* and *UHem-uHand*, we found similar dominant network patterns during both sessions 1 and 2. It was model 8 which was dominant with exceedance probability of (i) 0.821 for session 1 (ii) 0.534 for session 2 for *UHem-aHand* and (iii) 0.723 for session 1 and 0.542 for session 2 for *UHem-uHand*. Model 6 was the second best model for session 2 with exceedance probability of (i) 0.359 for *UHem-aHand* and (ii) 0.349 for *UHem-uHand*.

A decreasing exceedance probability occurred for model 8 during session 1 from 0.987 for *AHem-aHand* and 0.787 for *AHem-bHand* to 0.821 for *UHem-aHand* and further decreased to 0.723 for *UHem-uHand*. Similarly, an increase in exceedance probability for model 6 was found during session 2 from 0.175 for *AHem-aHand* and 0.174 for *AHem-bHand* to 0.359 for **UHem-aHand** and 0.349 for *UHem-uHand*.

The model exceedance probabilities of the optimal models for each analysis and session calculated using BMS approach are summarized in **Supplementary Table S2**.

### Effective Connectivity: Bayesian Model Averaging (BMA)

We used paired two-tailed *t-tests* to determine the significant endogenous and modulatory connections in both affected and unaffected hemisphere of motor network.

### *AHem-aHand* and *AHem-bHand*

**FIGURE 6:**
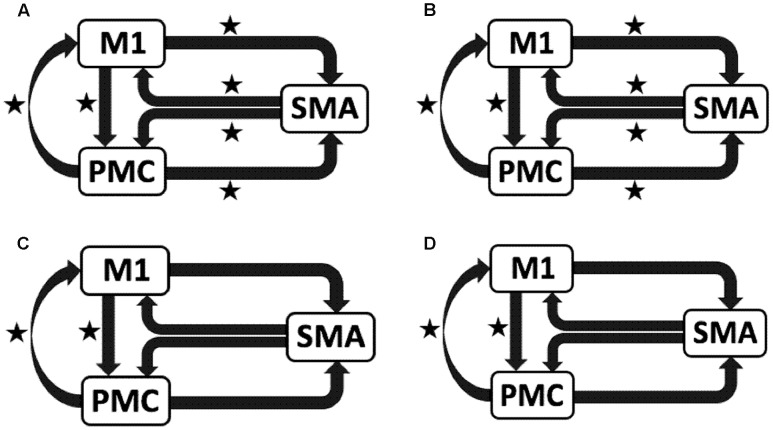
**Endogenous connectivity for affected hemisphere.** Endogenous connections for *AHem-aHand* (when the task was performed with only affected hand) and *AHem-bHand* (when the task was performed with both the hands: affected and unaffected) are shown for session 1 **(A,B)** and session 2 **(C,D)**. Here ^∗^ represents the connections which are significantly stronger (^∗^*p* < 0.001, *t*-test).

We reported significant (^∗^*p* < 0.001) endogenous connections (**Figure 6**) on the affected hemisphere when patients tapped with the affected hand (**Figure 6A**), i.e., *AHem-aHand*; both affected and unaffected hand (**Figure 6B**), i.e., *AHem-bHand* during session 1; affected hand (**Figure 6C**), i.e., *AHem-aHand* and both affected and unaffected hand (**Figure 6D**), i.e., *AHem-bHand* during session 2.

### *UHem-aHand* and *UHem-uHand*

**FIGURE 7:**
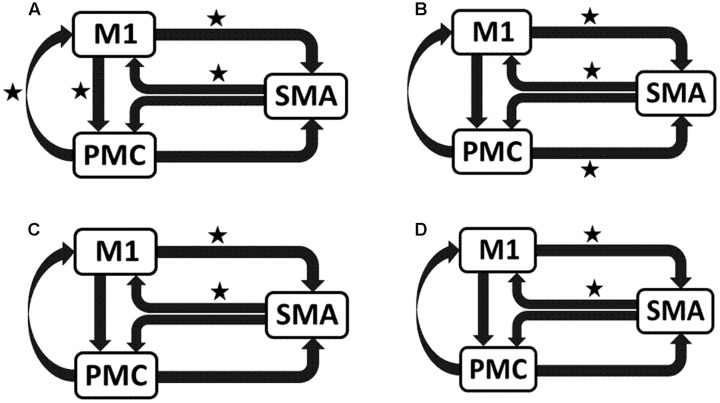
**Endogenous connectivity for unaffected hemisphere.** Endogenous connections for *UHem-aHand* (when the task was performed with affected hand) and *UHem-uHand* (when the task was performed with unaffected hand) are shown for session 1 **(A,B)** and session 2 **(C,D)**. Here ^∗^ represents the connections which are significantly stronger (^∗^*p* < 0.001, *t*-test).

We reported significant (^∗^p < 0.001) connections (**Figure 7**) on unaffected hemisphere when patients tapped with affected hand (**Figure 7A**), i.e., *UHem-aHand*; unaffected hand (**Figure 7B**), i.e., *UHem-uHand* during session 1; affected hand (**Figure 7C**), i.e., *UHem-aHand* and unaffected hand (**Figure 7D**), i.e., *UHem-uHand* during session 2.

When we compared individual connections for *AHem-aHand, UHem-aHand*, *and UHem-uHand* during session 2, two-tailed *t*-test showed that the connection between SMA and M1 for *UHem-aHand* and *UHem-uHand* was significantly different from *AHem-aHand* (*p* = 0.0443 and *p* = 0.0369 for SMA to M1 and M1 to SMA, respectively, for *AHem-aHand* versus *UHem-aHand* and *p* = 0.0453 and *p* = 0.0371 for SMA to M1 and M1 to SMA, respectively, for *AHem-aHand* versus *UHem-uHand* but there was no significant difference between *UHem-aHand* and *UHem-uHand* for any connection.

Hence, using the BMA approach, we found that the individual connectivity between motor areas did not change on the affected hemisphere whether patients tapped with only the affected hand or with both affected as well as their unaffected hand during both the sessions 1 and 2. We also found that the bidirectional connectivity between SMA and M1 was common between *UHem-aHand* and *UHem-uHand* for both the sessions 1 and 2. Also, the bidirectional connection between SMA and M1 was significantly stronger for *UHem-aHand* and *UHem-uHand* than *AHem-aHand*.

The data suggest that the connectivity pattern was identical between *AHem-aHand* and *AHem-bHand* and also there was more similarity of connectivity patterns between *UHem-aHand* and *UHem-uHand* than between *AHem-aHand* and *UHem-aHand* or between *AHem-aHand* and *UHem-uHand* during both the sessions 1 and 2. We did not find any modulatory connection that was significantly stronger (*p* < 0.05) for any analysis but we certainly noticed a trend for a modulatory connection (^∗∗^*p* < 0.1) from PMC to M1 for *UHem-aHand* and *UHem-uHand*, but not on affected hemisphere. Further, we also noticed that the individual significant connectivity differed when calculated a week apart. But *t*-tests showed that none of the connections differed significantly when compared for session 1 with session 2 for all the analyses.

Connectivity strength measures, standard deviation and significance level (^∗^*p* < 0.001 for endogenous connections, ^∗∗^*p* < 0.1 for modulatory connections) extracted using BMA for all the connections and for each analysis and each session are summarized in **Supplementary Table S3**.

### Effective Connectivity versus Clinical Scores

**FIGURE 8:**
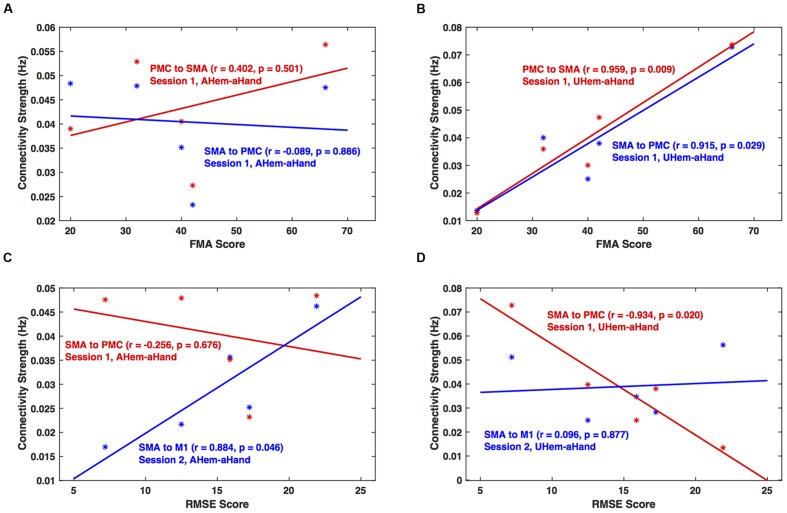
**Brain versus behavioral correlation.** Brain connectivity strength measures (in Hz) are plotted with the Fugl-Meyer Motor Assessment (FMA) scores **(A,B)** and root means square error (RMSE) **(C,D)** for *AHem-aHand* **(A,C)** and *UHem-aHand* **(B,D)**.

We calculated the correlation between clinical scores (FMA and RMSE) and connectivity strengths (in Hz) of all the connections for each analysis and session using Pearson’s linear correlation coefficient test. We found that for session 1, the correlation between connectivity strength between SMA and PMC and FMA score was not significant (*p* > 0.05) for *AHem-aHand* (**Figure 8A**). But there was a significant correlation (*p* < 0.05) for *UHem-aHand* (**Figure 8B**). The correlation between connectivity strength from SMA to PMC and from SMA to M1 and RMSE score was not significant (*r* = –0.256, *p* = 0.676) for AHem-aHand, session 1 and was positively significant (*r* = 0.884, *p* = 0.046) for *AHem-aHand*, session 2, respectively, (**Figure 8C**). Alternatively, the correlation between connectivity strength from SMA to PMC and from SMA to M1 and RMSE score was negative (*r* = –0.934, *p* = 0.020) for *UHem-aHand*, session 1 and was not significant (*r* = 0.096, *p* = 0.877) for *UHem-aHand*, session 2, respectively (**Figure 8D**). Here higher FMA scores and lower RMSE scores represent better patient performance and vice-versa.

Hence, by correlating individual connectivity strengths and clinical scores for *AHem-aHand* and *UHem-aHand* for both the sessions 1 and 2, we observed significant positive correlation between brain and behavior measures (FMA scores) for two connections for *UHem-aHand* during session 1. We also found that RMSE scores had no negative correlation with brain connectivity measures for *AHem-aHand* but there was negative correlation between RMSE scores and connectivity measures for *UHem-aHand*.

## Discussion

In this study, we explored and compared the role of the affected and unaffected hemisphere during hand motor execution tasks in chronic stroke survivors. We also performed the test–retest reliability of connectivity measures between motor-areas of both hemispheres. Comparing BMS results for *AHem-aHand* and *AHem-bHand*, we found that (a) the network pattern did not change on the affected hemisphere whether patients tapped with only the affected hand or with both their affected as well as their unaffected hand during both the sessions 1 and 2 and (b) the network pattern was unchanged when analyzed a week apart for both the *AHem-aHand* and *AHem-bHand*. Further, we found that when a person who has had a stroke tapped with their affected hand, the network connectivity pattern on their unaffected hemisphere dominated over the affected hemisphere. The network pattern resembled the form of their unaffected hemisphere when they tapped with their unaffected hand. This suggests that the so-called unaffected hemisphere of stroke survivors was influenced when they tapped with their affected hand. Additionally, these observed connectivity strength characteristics appear to be stable across two observations one week apart. These findings not only reflect the reliability of fMRI technique but also of the effective connectivity approach (DCM12). From brain versus behavior (FMA and RMSE) co-relation, we observed that the connectivity pattern on unaffected hemisphere more accurately reflected the connectivity measures than the connectivity pattern on affected hemisphere when stroke patients tapped with affected hand.

### Contribution of Unaffected and Affected Hemispheres

In this study, we found that the unaffected hemisphere better reflected the connectivity measures than affected hemisphere when a motor task was performed with affected hand, whereas the network pattern remained the same on the affected hemisphere independent of whether the task was performed with affected hand or with both hands. When correlating brain connectivity with measures of clinical motor behavior we found that during one of the sessions, the connectivity strength between SMA and PMC on the unaffected hemisphere was significantly (positively) correlated with FMA scores when patients performed the task with their affected hand. Also during one of the sessions, the connectivity strength from SMA to PMC on the unaffected hemisphere was found to be negatively correlated (*p* = 0.020) with RMSE scores when patients performed the task with affected hand. These correlations between brain networks and behavior measures reflect the fact that the connectivity pattern on unaffected hemisphere more accurately reflect the connectivity measures when patients tapped with their affected hand than the connectivity pattern of the affected hemisphere when patients tapped with their affected hand. These findings also confirmed our findings from both BMS and BMA approaches that it is the unaffected hemisphere of stroke survivors that better reflects the connectivity pattern than the affected hemisphere during a motor execution task.

In a study of eight well-recovered stroke patients, Riecker et al. (2010) studied the contribution of affected and unaffected hemisphere during motor-recovery. The authors found a linear correlation between hemodynamic responses from the affected as well as the unaffected premotor motor cortex and sensorimotor cortex and frequency of finger movements. Although this study supported the idea of bi-hemispheric recruitment in stroke survivors, their data was confounded by the use of well-recovered stroke survivors. Additionally, the authors did not include a direct comparison of the involvement of affected and unaffected hemispheres. Using transcranial direct current stimulation, Fregni et al. (2005) explored the significance of the unaffected hemisphere for a better motor recovery. In this study, it was suggested that increased activity in the affected hemisphere can enhance the motor recovery but excessive activity in the unaffected hemisphere may provide an inadequate environment for motor recovery. So a properly balanced bi-hemispheric modulation of brain tissues was recommended in order to effectively promote the motor recovery. Reorganization of motor-output was also explored for the unaffected hemisphere of stroke patients (Netz et al., 1997). In this study, the motor-outputs from the unaffected hemisphere were significantly changed after stroke, although the brain activation was not correlated with clinical improvement. This study supported the idea of plastic changes in the unaffected hemisphere during motor output organization. In a recent diffusion tensor imaging (DTI) study (Jang et al., 2016), fractional anisotropy (FA) and apparent diffusion coefficient (ADC) values of the corticospinal tract (CST) were reported to be unchanged for the unaffected hemisphere but fiber volume of CST in the unaffected hemisphere was found to be increased (Kwak et al., 2010; Jang et al., 2016). There are very few studies, which report a decrease in network activity on the unaffected hemisphere. Dong and colleagues found an increase in cortical activation of M1 in the unaffected hemisphere but a correlation was also observed between decreased cortical activation and restoration of motor function (Dong et al., 2006). On the other hand, less affected brain side was also reported to create an abnormal activation pattern which limits the normal activation environment in the motor-network (Takeuchi and Izumi, 2012). In order to limit this maladaptive neural plasticity, specific rehabilitation techniques such as noninvasive brain stimulation were also proposed (Fregni et al., 2005; Takeuchi and Izumi, 2012).

Furthermore, comparing the contribution of both hemispheres, our study demonstrated the dominance of unaffected hemisphere over affected hemisphere when the task was performed with the affected hand. We also found that the network pattern of the unaffected hemisphere when the task was performed with the affected hand resembled the network pattern of the unaffected hemisphere when the task was performed with the unaffected hand. Hence, these findings suggested that the dominance of unaffected hemisphere might reflect a compensatory hub of connections after stroke. These results also indicate a complex functional behavior of motor networks in stroke patients. Results from previous studies are in accordance with our findings whereas a few studies reported controversial outcomes. While behavioral improvement were found to be in close association with sensorimotor networks consisting of medial PMC, lateral PMC, primary motor cortex and primary somatosensory cortices in the affected hemisphere (Pineiro et al., 2002; Loubinoux et al., 2003; Calautti et al., 2007; Loubinoux, 2007), functional reorganization within unaffected hemisphere was found to be supporting motor recovery (O’Shea et al., 2007; Riecker et al., 2010; Rehme et al., 2011). Besides these reports, in a study of five patients, increased cortical activation was reported in the unaffected hemisphere during movement of the affected hand at less than 25 days after onset of the stroke but the activation was found to be normal at 35 days after onset (Takeda et al., 2007). A more detailed description of the changes in functional network after stroke was provided by Li et al. (2014) where they reported that some of the damaged connections of functional network could be compensated by new indirect connections or circuits produced after stroke (Li et al., 2014). Hence, these studies confirmed our findings that the unaffected hemisphere always had a role to play after stroke by dominating and compensating the role of affected hemisphere.

### Test–Retest Reliability

After comparing the exceedance probabilities of Bayesian models after a week apart, we found that the dominant network was consistent for (i) the affected hemisphere when the task was performed with either the affected hand or with both the hands and (ii) the unaffected hemisphere when the task was performed with either the affected hand or unaffected hand. Furthermore, by comparing the individual connectivity strengths calculated using the BMA approach, we did not find any connections, which differ significantly measured one week apart.

From several years, fMRI has emerged as one of the most popular and commonly used technique to study brain activations and for advanced-level connectivity analysis. Studies like fMRI reliability with time play a significant role in identifying an effective and reliable stroke rehabilitation technique among several. Previously, several studies reported the reliability of BOLD fMRI signals over time in healthy people during a variety of tasks such as cognitive, sensorimotor, working memory, motor-imagery, and execution tasks (Loubinoux et al., 2001; Aron et al., 2006; Lim et al., 2007; Yoo et al., 2007; Kimberley et al., 2008a,b; Caceres et al., 2009). Several other studies on healthy controls even reported very high variability in the magnitude and spatial extent of activations when comparison was done between sessions (Miki et al., 2000; Loubinoux et al., 2001; Liu et al., 2004). These studies suggested that the reliability of fMRI is yet to be confirmed even for healthy controls. It can be imagined how controversial fMRI results could be for reliability studies of impaired individuals and for people suffering from more complex brain disorders. Further, network and individual connectivity analysis might further add variability to the connectivity results and can originate more controversial findings based on fMRI technique.

Further to translate neuronal activity to BOLD signal, neuronal model implemented in DCM is considered as one of the most appropriate models as it is more flexible with the shape of hemodynamic response function (HRF) (Schuyler et al., 2010). Schuyler et al. (2010) also reported that brain activation and its interactions were more reliable when connectivity information was considered in comparison to when only effects of general linear model (GLM) were considered. DCM was also considered to be very sensitive to the nuances of fMRI signal changes and a fair to excellent scan-rescan DCM reliability was reported (Schuyler et al., 2010). However, the test–retest reliability of DCM for fMRI was again in question recently when different versions of DCM were tested. Classical DCM in SPM5 was found be more reliable than DCM10 in SPM8 for fMRI (Frässle et al., 2015). But in another study of face perception network by Frässle et al. (2016), stable conventional activity and effective connectivity measures were obtained using BMS and parameter estimation approach.

## Conclusion

Results of the present study uncovered (i) an important brain-behavior relationship between the connectivity in the unaffected brain hemisphere and the motor behavior of the affected hands in stroke patients and (ii) the test–retest reliability of fMRI and effective connectivity approach. Findings reported in this study strengthened our understanding of stroke conditions and brain plasticity in stroke survivors. We believe that such studies play a crucial role identifying an effective stroke rehabilitation therapy. Future studies with a larger sample size and involvement of age-matched healthy controls would further enhance the importance of unaffected hemisphere and its role while treating stroke patients.

## Author Contributions

Conceived and designed the experiments: DW, GJ, AB. Performed the experiments: SB, DW, AB. Analyzed the data: SB, SH. Wrote the paper: SB, SH, MD, GJ, AB.

## Conflict of Interest Statement

The authors declare that the research was conducted in the absence of any commercial or financial relationships that could be construed as a potential conflict of interest.

## Footnotes

1 http://www.fil.ion.ucl.ac.uk/spm/software/spm12/

## Supplementary Material

**TABLE S1 | Demographics, stroke locations and behavioral scores for stroke survivors.**

**TABLE S2 | Optimal model selection.** Model excedance probabilities of optimal models for each case and session calculated using Bayesian model selection (BMS) approach are summarized.

**TABLE S3 | Bayesian model averaging (BMA) parameters.** Connectivity strength measures, standard deviation and significance level (^∗^*p* < 0.001 for endogenous connections, ^∗∗^*p* < 0.1 for modulatory connections, *t*-test) extracted using Bayesian model averaging (BMA) for all the connections and for each case and each session are summarized.

**FIGURE S1 |*AHem-aHand, AHem-bHand, UHem-aHand*, and *UHem-uHand*.** Here we summarize all the four cases of connectivity analysis. **(A–D)**: Here ‘affected’ hemisphere, colored as ‘red’ is under study when stroke survivors performed the task with affected hand i.e., either right affected hand **(A)** or left affected hand **(B)** (*AHem-aHand*) and with both the hands i.e., with either right affected and left unaffected **(C)** or left affected and right unaffected **(D)** (*AHem-bHand*). **(E,F)**: Here ‘unaffected’ hemisphere, colored as ‘gray’ is under study when stroke survivors performed the task with affected hand i.e., either right affected hand **(E)** or left affected hand **(F)** (*UHem-aHand*). **(G,H)**: Here ‘unaffected’ hemisphere, colored as ‘gray’ is under study when stroke survivors performed the task with unaffected hand i.e., either left unaffected hand **(G)** or right unaffected hand **(H)** (*UHem-uHand*).

## Abbreviations

*AHem-aHand*: Affected hemisphere, affected hand
*AHem-bHand*: Affected hemisphere, both hands (affected and unaffected)
*UHem-aHand*: Unaffected hemisphere, affected hand
*UHem-uHand*: Unaffected hemisphere, unaffected hand
