# Supplementary material for: Dominance of the Unaffected Hemisphere Motor Network and Its Role in the Behavior of Chronic Stroke Survivors
Source: Front Hum Neurosci. 2016 Dec 27;10:650. doi: 10.3389/fnhum.2016.00650 (PMC5186808; doi:10.3389/fnhum.2016.00650)
Supplement: Supplementary file 1 [file Table_1.docx]

| Participant | Age  (Years) | Sex | Time post stroke (months) | Stroke Location | Baseline FMA Score | Baseline RMSE Score |
| --- | --- | --- | --- | --- | --- | --- |
| 1 | 61 | F | 18.6 | Left subcortical MCA territory | 66 | 7.2 |
| 2 | 57 | M | 60 | Right MCA territory | NA | NA |
| 3 | 74 | F | 24 | Right subcortical MCA | NA | NA |
| 4 | 62 | M | NA | Left cortical MCA | NA | NA |
| 5 | 80 | M | 30.2 | Right cortical MCA territory with subcortical sparing | 42 | 17.23 |
| 6 | 50 | M | 24 | Left subcortical MCA territory | 32 | 12.48 |
| 7 | 54 | M | 10.1 | Right cortical MCA territory | 20 | 21.91 |
| 8 | 56 | M | 12.9 | Left cortical MCA territory with subcortical sparing | 40 | 15.9 |

**Supplementary Table S1**

**FMA:** Fugl-Meyer Motor Assessment (FMA); **RMSE:** Root mean square error; **NA:** Not Available
