# Supplementary material for: Dominance of the Unaffected Hemisphere Motor Network and Its Role in the Behavior of Chronic Stroke Survivors
Source: Front Hum Neurosci. 2016 Dec 27;10:650. doi: 10.3389/fnhum.2016.00650 (PMC5186808; doi:10.3389/fnhum.2016.00650)
Supplement: Supplementary file 2 [file Table_2.docx]

**Supplementary Table S2**

| **Cases** | **Optimal model selection** | | | |
| --- | --- | --- | --- | --- |
|  | **Session 1** | | **Session 2** | |
|  | ***Model*** | ***E.P.*** | ***Model*** | ***E.P.*** |
| *AHem-aHand* | 8 | 0.987 | 8  6 | 0.787  0.175 |
| *AHem-bHand* | 8 | 0.984 | 8  6 | 0.786  0.174 |
| *UHem-aHand* | 8 | 0.821 | 8  6 | 0.534  0.359 |
| *UHem-uHand* | 8 | 0.723 | 8  6 | 0.542  0.349 |

E.P.: Exceedance probability
