# Supplementary material for: Dominance of the Unaffected Hemisphere Motor Network and Its Role in the Behavior of Chronic Stroke Survivors
Source: Front Hum Neurosci. 2016 Dec 27;10:650. doi: 10.3389/fnhum.2016.00650 (PMC5186808; doi:10.3389/fnhum.2016.00650)
Supplement: Supplementary file 3 [file Table_3.docx]

| Cases | Connections | Connectivity parameters | | | | | |
| --- | --- | --- | --- | --- | --- | --- | --- |
|  |  | Session 1 | | | Session 2 | | |
|  |  | *Mean* | *S.D* | *p-value* | *Mean* | *S.D.* | *p-value* |
|  | *Endogenous* | | | | | | |
| Case 1a | PMC→M1 | 0.034 | 0.010 | 0.000^*^ | 0.037 | 0.008 | 0.000^*^ |
|  | SMA→M1 | 0.027 | 0.012 | 0.000^*^ | 0.025 | 0.007 | 0.001 |
|  | M1→PMC | 0.037 | 0.008 | 0.000^*^ | 0.037 | 0.006 | 0.000^*^ |
|  | SMA→PMC | 0.038 | 0.010 | 0.000^*^ | 0.050 | 0.006 | 0.003 |
|  | M1→SMA | 0.028 | 0.008 | 0.000^*^ | 0.028 | 0.006 | 0.001 |
|  | PMC→SMA | 0.041 | 0.008 | 0.000^*^ | 0.054 | 0.007 | 0.002 |
|  | *Modulatory* | | | | | | |
|  | PMC→M1 | 0.010 | 0.010 | 0.176 | 0.008 | 0.008 | 0.122 |
|  | SMA→M1 | -0.001 | 0.012 | 0.735 | 0.005 | 0.007 | 0.403 |
|  | SMA→PMC | 0.037 | 0.010 | 0.118 | 0.001 | 0.006 | 0.822 |
| Case 1b | *Endogenous* | | | | | | |
|  | PMC→M1 | 0.035 | 0.010 | 0.000^*^ | 0.036 | 0.008 | 0.000^*^ |
|  | SMA→M1 | 0.027 | 0.012 | 0.000^*^ | 0.025 | 0.007 | 0.001 |
|  | M1→PMC | 0.036 | 0.008 | 0.000^*^ | 0.037 | 0.006 | 0.000^*^ |
|  | SMA→PMC | 0.038 | 0.010 | 0.000^*^ | 0.049 | 0.006 | 0.003 |
|  | M1→SMA | 0.028 | 0.008 | 0.000^*^ | 0.028 | 0.006 | 0.001 |
|  | PMC→SMA | 0.041 | 0.008 | 0.000^*^ | 0.054 | 0.007 | 0.002 |
|  | *Modulatory* | | | | | | |
|  | PMC→M1 | 0.011 | 0.010 | 0.143 | 0.008 | 0.008 | 0.1134 |
|  | SMA→M1 | -0.003 | 0.012 | 0.467 | 0.005 | 0.007 | 0.4145 |
|  | SMA→PMC | 0.037 | 0.010 | 0.117 | 0.001 | 0.006 | 0.8045 |
| Case 2 | *Endogenous* | | | | | | |
|  | PMC→M1 | 0.030 | 0.009 | 0.000^*^ | 0.035 | 0.006 | 0.002 |
|  | SMA→M1 | 0.026 | 0.013 | 0.000^*^ | 0.037 | 0.006 | 0.000^*^ |
|  | M1→PMC | 0.031 | 0.006 | 0.000^*^ | 0.039 | 0.005 | 0.002 |
|  | SMA→PMC | 0.045 | 0.011 | 0.006 | 0.055 | 0.006 | 0.004 |
|  | M1→SMA | 0.028 | 0.006 | 0.000^*^ | 0.040 | 0.005 | 0.000^*^ |
|  | PMC→SMA | 0.047 | 0.008 | 0.003 | 0.049 | 0.006 | 0.001 |
|  | *Modulatory* | | | | | | |
|  | PMC→M1 | 0.004 | 0.009 | 0.064^**^ | 0.009 | 0.006 | 0.107 |
|  | SMA→M1 | -0.001 | 0.013 | 0.665 | 0.000 | 0.006 | 0.890 |
|  | SMA→PMC | 0.007 | 0.011 | 0.370 | 0.001 | 0.006 | 0.785 |
| Case 3 | *Endogenous* | | | | | | |
|  | PMC→M1 | 0.042 | 0.005 | 0.002 | 0.034 | 0.006 | 0.002 |
|  | SMA→M1 | 0.033 | 0.010 | 0.000^*^ | 0.037 | 0.006 | 0.000^*^ |
|  | M1→PMC | 0.042 | 0.004 | 0.001 | 0.039 | 0.005 | 0.002 |
|  | SMA→PMC | 0.054 | 0.009 | 0.001 | 0.055 | 0.006 | 0.004 |
|  | M1→SMA | 0.035 | 0.004 | 0.000^*^ | 0.039 | 0.005 | 0.000^*^ |
|  | PMC→SMA | 0.055 | 0.004 | 0.000^*^ | 0.049 | 0.006 | 0.001 |
|  | *Modulatory* | | | | | | |
|  | PMC→M1 | 0.010 | 0.005 | 0.0518^**^ | 0.009 | 0.006 | 0.108 |
|  | SMA→M1 | 0.007 | 0.010 | 0.4642 | 0.000 | 0.006 | 0.908 |
|  | SMA→PMC | 0.009 | 0.009 | 0.3570 | 0.001 | 0.006 | 0.788 |

S.D.: Standard Deviation; ^*^p < 0.001 for endogenous connections and ^**^p < 0.1 for modulatory connections.

**Supplementary Table S3**
