# Supplementary figures and images for: Dominance of the Unaffected Hemisphere Motor Network and Its Role in the Behavior of Chronic Stroke Survivors
Source: Front Hum Neurosci. 2016 Dec 27;10:650. doi: 10.3389/fnhum.2016.00650 (PMC5186808; doi:10.3389/fnhum.2016.00650)

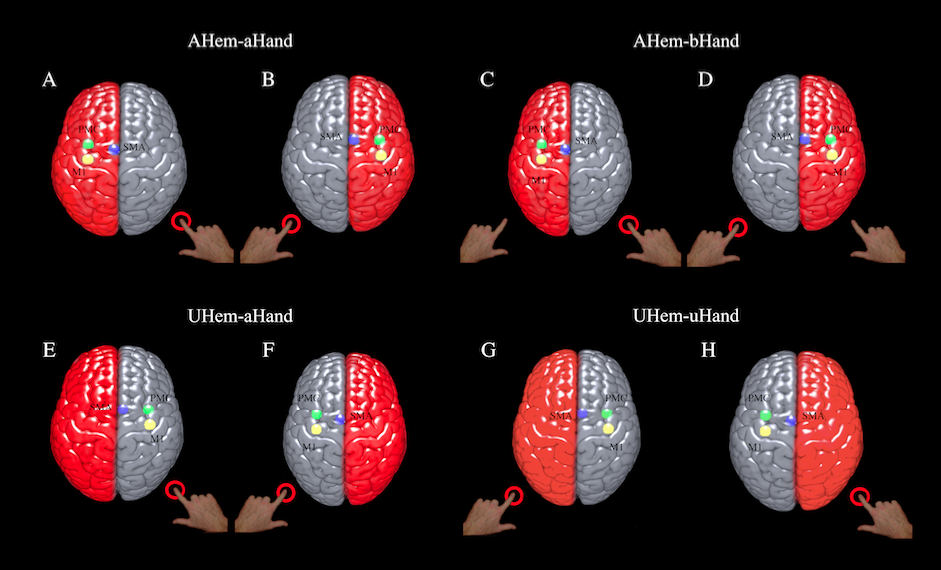

Supplement: Supplementary file 4 [file Image_1.png]
